# The revised Psychosis Attachment Measure: further psychometric evidence

**DOI:** 10.1007/s00127-024-02624-2

**Published:** 2024-03-19

**Authors:** Miranda Justo-Nunez, Lydia Morris, Katherine Berry

**Affiliations:** 1 Division of Psychology and Mental Health, University of Manchester, Oxford Road, Manchester, M13 9PL UK; 2 Department of Research and Innovation, Greater Manchester Mental Health NHS Foundation Trust, Oxford, Manchester, M13 9PWL UK

**Keywords:** Self-report, Psychometric, Questionnaire, Validity, Reliability

## Abstract

**Purpose:**

Disorganised attachment is a key concept in understanding the development of psychosis. However, existing questionnaires of adult attachment do not adequately measure this construct hindering future research into the psychosocial causes of psychosis. The most widely measure of adult attachment in people experiencing psychosis is the Psychosis Attachment Measure (PAM). The measure has recently been revised to include disorganised attachment items. This study develops previous research by providing a rigorous examination the psychometric properties of the revised questionnaire (PAM-R).

**Methods:**

A total of 407 participants with self-reported experiences of psychosis completed a battery of questionnaires which included the PAM-R and other measures which were conceptually related to the concept of disorganised attachment.

**Results:**

Confirmatory factor analysis (CFA) indicated a three-factor solution with factors corresponding to anxious, avoidant, and disorganised attachment. The majority of the fit statistics were acceptable with the exception of the RMSEA statistic. Internal consistency and test–retest reliability were good for all subscales. The disorganised subscale correlated in expected directions with other measures of attachment, dissociation, trauma, and psychotic experiences.

**Conclusion:**

The PAM-R is a valid and reliable measure of adult attachment. It is a practical assessment tool for clinicians and researchers to measure insecure and disorganised attachment patterns that is acceptable to people experiencing psychosis.

**Supplementary Information:**

The online version contains supplementary material available at 10.1007/s00127-024-02624-2.

## Introduction

Psychosis is a serious mental health issue affecting 20 million people across the globe, and is associated with experiences such as hallucinations, delusions, and paranoia [1]. The proposed biological mechanisms underpinning psychosis have historically dominated research efforts. However, there has been a growing body of evidence demonstrating the link between childhood trauma and the nature and severity of psychotic symptoms [2–4], as well as the prevalence of interpersonal difficulties amongst this group [5]. This has highlighted that further exploration of psychosocial processes is a necessary, and promising, line of enquiry to better understand the onset and maintenance of psychosis [6].

Attachment theory has been identified as one psychological model which may help to delineate the relational component of this condition [7]. Attachment theory argues that our early relationships lead us to form “internal working models” from infancy, which include dynamic representations of the self, others, and the self within relationships [8]. This allows individuals to make sense of and interpret interpersonal interactions, informing their own behaviour within relationships and ultimately leading to the development of their attachment style [9]. Infants tend to form a “secure” attachment style when caregivers are emotionally attuned and available [10]. Caregivers provide a source of comfort and safety, which enables them to explore and appropriately manage distressing situations [11]. Where there are protracted difficulties in key relationships, particularly with respects to the caregiver’s sensitivity to infant distress [12], children a more likely to be insecurely attached, and develop an anxious, avoidant, or disorganised attachment pattern [13, 14].

Attachment styles appear to remain moderately stable from childhood to adulthood [15, 16], however can be subject to change, especially following significant adverse life events [17, 18], or exposure to positive alternative support figures [19]. In adulthood, whilst secure attachment has been associated with improved emotional regulation, social competence, and self-esteem [20, 21], insecure attachment styles have been linked with poorer mental health outcomes, relationship satisfaction, and resilience in coping with stressors [22–24].

Childhood trauma (including sexual, physical, and emotional abuse, parental death, neglect, and bullying) inherently threatens the formation of secure bonds. Given the link between trauma and psychosis, it is perhaps unsurprising that the prevalence of insecure attachment styles has been found to be significantly higher amongst this population compared to non-clinical samples [25, 26]. As such, it has been argued that attachment insecurity may act as a mediating factor in the relationship between childhood trauma and the onset of psychosis [7]. Indeed, there is a growing body of evidence in support of this hypothesis, illustrating the mediating role of attachment with respect to a wide range of psychotic phenomena, including paranoia [27] and voice-hearing [28]. There is a related body of research investigating factors which mediate the association between attachment and psychosis including negative beliefs, poor emotional regulation, poor cognitive diffusion [29], resilience [30], and experiential avoidance [31].

More recently, the concept of disorganised attachment has attracted substantial interest within psychosis research. This attachment pattern was first documented by Main and Solomon [14], who identified a subgroup of infants that appeared disoriented or demonstrated contradictory behaviour upon being reunited with their caregiver. It has been hypothesised that this develops when infants repeatedly experience the paradoxical situation whereby their caregiver is both the person that they turn to in times of distress, and the source of their fear [32]. The infant’s “fright without solution” then manifests in simultaneous or sequential attempts to approach and flee the caregiver, freezing, or dissociation [33]. In adulthood, the fear of both intimacy and abandonment underpins similar conflictual patterns of behaviour, accompanied by feelings of confusion and mistrust towards others [34]. This attachment pattern appears to predispose individuals to dissociative experiences [35], which is proposed to be a central process that contributes to development of voice-hearing [36]. Crucially, disorganised attachment has been associated with higher incidents of sexual and physical abuse in people living with psychosis, and more severe hallucinations and delusions, compared to other attachment styles [37]. These findings further highlight the potential for attachment theory to enrich current conceptualisations of psychosis and trauma, and identify areas for intervention. In turn, this requires valid and reliable measures of attachment that can be readily administered in both research and clinical settings.

Despite the abundance of self-report measures of attachment, a recent review conducted by Pollard et al. [38] suggests that many lack a robust evidence base or do not adequately capture the concept of disorganised attachment. For example, several self-report measures include a measure of fearful attachment, which is characterised by individuals vacillating between approach and avoidance behaviours in relationships and is thought to conceptually similar to but not equivalent to disorganised attachment as described in infancy. Similarly, many measures (i.e., Adult Disorganised Attachment Scale [ADA] [34]; The Psychological Treatment Inventory—Attachment Styles Scale [PTI-ASS] [39]) are designed with romantic relationships in mind. These items may not be as accessible for individuals with psychosis, who can often struggle to form and maintain close relationships [40]. The Psychosis Attachment Measure (PAM) is the most widely used adult attachment measure with people experiencing psychosis and assess avoidance and anxious attachment. Pollard et al. [41] recently adapted the measure to include items to capture disorganised attachment. The authors subjected 28 items which included 16 items in the original scale and 12 disorganised items to an EFA. They provided encouraging preliminary evidence regarding the psychometric properties of the PAM-R, although the authors’ results suggested removal of five items (including two of the original avoidance items) resulting in a 23-item measure. The present study aimed to build on the work of Pollard et al. to confirm the structural dimensionality, reliability, and validity of the PAM-R in an independent sample. Confirmatory factor analysis is an important step in measure development which aims to assess the stability of factor structures in new samples.

The following study hypotheses were developed in line with the Consensus-based Standards for the selection of health Measurement Instruments’ (COSMIN) criteria for good measurement properties [42].

1. Confirmatory Factor Analysis will indicate a three-factor model of the PAM-R, according to multiple fit indices.
2. Cronbach’s alphas used to determine internal consistency will be > 0.7 for all subscales.
3. Test–retest reliability, as measured by Intraclass Correlation Coefficients (ICCs), will exceed 0.75 for all subscales.
4. There will be a moderate positive association (correlation coefficients > 0.3) between the disorganised subscale and other measures which are conceptually related to this attachment style, including measures of trauma, dissociation, and psychotic experiences.
5. A large positive relationship (> 0.5) will be observed between the disorganised factor of the PAM-R and the corresponding subscale of other measures of attachment.

## Method

### Participants

Participants were eligible to take part in the study providing that they (i) were aged 18 or over, (ii) had a self-reported diagnosis of a psychotic disorder or had received treatment for experiences related to psychosis, and (iii) were proficient in English. Participants were recruited online between January 2021 and December 2021, and completed all measures online. These data were combined with an existing data set collected as part of two previously published doctoral projects [43, 44]. The eligibility criteria, measures, and procedures, for this existing dataset, can be assumed to be the same unless otherwise stated in this paper.

### Measures

#### Demographic questionnaire

This collected information about the participant’s gender, age, ethnicity, sexual orientation, relationship status, academic qualifications, and employment status. Questions around participants’ psychiatric diagnoses and experiences of mental health support relating to symptoms associated with psychosis were also included.

#### The Brief Betrayal Trauma Survey (BBTS)

The BBTS [45] is a 12-item self-report measure designed to assess exposure to trauma in childhood and adulthood. Participants were asked to rate their experience of adverse life events on a 3-point Likert scale (1—“never”; 2—“one or two times”; 3—“more than that”), before and after the age of 18. Freyd et al. [46] outline that items can also be grouped according to the level of betrayal: “high” (physical, emotional, or sexual abuse by someone close), “medium” (witnessing a traumatic event involving someone close or experiencing abuse perpetrated by someone more relationally distant), or “low” (witnessing a traumatic event involving someone less close, natural disasters, or accidents). Studies have indicated that the BBTS has good test–retest reliability [45] and construct validity [47]. Internal consistency within the present sample was *α* = 0.935 (before 18) and *α* = 0.835 (after age 18).

#### The Community Assessment Psychic Experiences—42 (CAPE)

The CAPE [48] is a 42-item self-report measure assessing positive and negative psychotic symptoms and depressive symptoms. Only the positive symptom subscale (20 items) was included within this study. Participants indicated what percentage of the time they experience the symptoms described in each question (0%—Never; 100%—Always). Good psychometric properties have been established for the CAPE, within both clinical and non-clinical samples [48, 49]. Internal consistency in this study was *α* = 0.795.

#### Dissociative Experiences Scale (DES-II)

DES-II [50] is a 28-item self-report measure assessing experiences of amnesia, depersonalisation, derealisation, and absorption. Participants were required to rate the extent to which they felt the item applied to their experiences, from 0 to 100% (0%—Never; 100%—Always). The DES-II is the most widely used measure of dissociative experiences, with numerous studies indicating good reliability and validity in both clinical and non-clinical samples [51]. Internal consistency in this sample was excellent (*α* = 0.944).

#### The Psychological Treatment Inventory-Attachment Styles Scale (PTI-ASS)

The PTI-ASS [39] is a 22-item self-report measure of all four adult attachment styles. The “unresolved” subscale provides a measure of disorganised attachment but like most measures of adult attachment the measure focussed on perceptions of oneself in the context of romantic relationships. Participants were required to indicate their level of agreement with each item, using a five-point Likert scale (1—“not at all”; 2—“somewhat”; 3—“moderately”; 4—“a good deal”; “very much”). Psychometric evaluation of the PTI-ASS provided evidence for its structural validity and internal consistency [36]. Internal consistency in the present sample was *α* = 0.73. The PTI-ASS was the only measure that was not administered in either Degnan et al. [44] nor Humphrey et al.’s [44] studies.

#### The Relationship Questionnaire (RQ)

The RQ [52] is a self-report measure designed to measure the four adult attachment styles. It consists of four paragraphs and respondents are required to rate to what extent they reflect their general relationship style, using a seven point Likert Scale (1—Disagree strongly; 7—Agree strongly). The “fearful” subscale is often considered as a proxy measure of disorganised attachment, but this has been contested in the literature [38]. The RQ is a widely used self-report measure of attachment, and reasonable psychometric properties have been reported [53].

#### The Revised Psychosis Attachment Measure (PAM-R)

The PAM-R [41] is 23-item self-report measure of anxious, avoidant, and disorganised attachment in psychosis. Participants were asked to rate to what extent each statement reflected how they relate to key people in their life, using a four-point Likert scale (“not at all”; “A little”; “quite a bit”; “very much”). Pollard et al. [41] reported promising psychometric properties in their initial validation study, including excellent test–retest reliability and preliminary evidence for the measure’s three-factor structure.

### Procedure

All procedures were then approved by The University of Manchester’s University Research Ethics Committee (Ref: 2020-10240-17162), which included permission to utilise the data from the previous studies. Advertisement materials were developed and were posted on social media platforms (Facebook, Twitter, Reddit, and Instagram). Participants were also recruited via mental health charities and peer support groups, who disseminated the study information via their social media accounts, website, or email lists where appropriate. The study link provided information about the study. Once participants provided their consent to participate, they were directed to the demographics form. The subsequent measures were presented in a random order to reduce order effects; however, the BBTS was placed in the middle to minimise participant distress. When the questionnaires were completed, participants were asked whether they could be contacted again in 2 weeks to complete the PAM-R. This step was not included in the previous studies [43, 44]. Participants were then presented with a debrief sheet, and were given the option to provide their email if they wanted to receive a summary of research findings and/or be entered into a prize draw. The participants who opted to complete the PAM-R 2 weeks later also completed this online, following the same format.

### Data analysis

Descriptive statistics, and tests of reliability and construct validity were carried out using IBM SPSS Statistics Version 25 [54]. The quality and distribution of the combined data was assessed first, which indicated that missing data were very low and that most variables were not normally distributed (Table 2). Accordingly, missing data were pro-rated with the median. BBTS responses were analysed according to age group (before 18 and after 18) and level of betrayal (high, medium, and low) as both factors were considered to be pertinent to the development of attachment styles. For construct validity, Spearman’s rank-order correlations were carried out as the data were not normally distributed for most measures. Confirmatory factor analysis (CFA) was conducted using the structural equation modelling module of JASP (version 0.16.00) [55], which is based on the R-package lavaan [56]. Diagonally Weighted Least Squares with robust error calculations was chosen as the estimation method, as this approach has been shown to perform well with ordinal data [57]. Model fit was evaluated using the Comparative Fit Index (CFI), Root-Mean-Square Error of Approximation (RMSEA), the Tucker-Lewis Index (TLI), and the Goodness-of-Fit Index (GFI). Schermelleh-Engel et al. [58] noted that acceptable model fit is indicated by CFI ≥ 0.95, RMSEA ≤ 0.08, TLI ≥ 0.95, and GFI ≥ 0.90.

## Results

The new data collected were combined with the previous datasets; this was considered appropriate given the inclusion criteria and recruitment methods were the same, and to achieve the sample size required for factor analysis. The results reported are based on the combined dataset unless otherwise stated. A total of 466 participants completed the demographic questionnaire; however, 59 individuals did not go on to provide responses for any of the study measures and were therefore excluded from the analysis. The final sample size across therefore 407.

### Sample characteristics

Demographic and clinical characteristics are presented in Table 1. The number of men and women was comparable; the majority identified as white and were aged between 18 and 34 years. Almost all participants reported that they had received a psychiatric diagnosis relating to psychosis, and most had a history of antipsychotic medication and/or mental health support (e.g., through a community mental health team).

**Table 1:** Combined sample demographic information and clinical characteristics

Characteristic	*N*	%
Sex		
Female	187	45.9
Male	170	41.8
Prefer to self-describe	45	11.1
Prefer not to say	5	1.2
Age		
18–24	158	38.8
25–34	137	33.7
35–44	56	13.8
45–54	28	6.9
55–64	15	3.7
65–74	8	2
75 +	3	.7
Prefer not to say/did not respond	2	.4
Ethnicity		
White British	164	40.3
White other	177	43.5
Mixed heritage	22	5.4
Asian	4	1
Black	2	.4
Chinese	8	2
Other ethnic background	26	6.4
Prefer not to say	4	1
Nationality		
British	161	39.6
American	128	31.4
European country	46	11.3
Canadian	22	5.4
Australian	17	4.2
Latin American country	12	3
Other nationality	18	4.4
Did not respond	3	.7
Sexual orientation		
Heterosexual	178	43.7
Gay or Lesbian	51	12.5
Bisexual	121	29.7
Other	45	11.1
Prefer not to say	12	2.9
First language		
English	345	84.7
Other	59	14.5
Prefer not to say/did not respond	3	.7
Marital status		
Never married and never registered in a civil partnership	313	76.9
Married	61	15.0
Separated, or previously married or in a civil partnership	30	7.4
Prefer not to say	3	.7
Highest qualification		
Higher qualification (e.g., degree, teaching, NVQ Level 4)	204	50.2
High school/A-level	99	24.3
Other qualifications	44	10.7
No qualifications	45	11.1
Prefer not to say	15	3.7
Employment status		
Employed or self-employed	147	36.1
Unemployed/receipt of sickness or disability allowance	122	30
Full-time education	100	24.6
Retired/looking after the family or home/other	36	8.8
Prefer not to say	2	.5
Self-report psychiatric diagnosis^a^		
Report ever receiving a psychiatric diagnosis	379	93.1
Schizophrenia/paranoid schizophrenia	92	22.6
Schizoaffective	115	28.3
Schizophreniform	9	2.4
Depression with psychotic features	137	33.7
Delusional disorder	12	2.9
Bipolar with psychotic features	106	26
Brief psychotic disorder	46	11.3
Any other disorder which includes psychotic experiences	89	21.9
Treatment		
History of antipsychotic medication	275	67.6
History of mental health support (e.g., CMHT or EIS)	359	88.2
Previous inpatient experience	266	65.3
Current mental health support (e.g., CMHT or EIS)	133	32.7
Current inpatient	1	.2

^a^ Participants could select more than one diagnosis; *CMHT* Community Mental Health Team, *EIS* Early Intervention Service

Descriptive statistics for the study measures can be found in Table 2. Of the 360 participants who completed the BBTS “Before 18” scale, 81.1% reported having experienced at least one trauma and 70.3% reported a “high” betrayal trauma, whereby they were physically, psychologically, or sexually abused by someone that they considered themselves to be very close to. A total of 74.4% stated that they had experienced a traumatic event after the age of 18, with 63.9% endorsing items that were indicative of “high” betrayal. Mean scores for the DES-II exceeded 30, the threshold that is considered to denote “clinically significant” levels of dissociation [50].

**Table 2:** Descriptive statistics for study measures

Measure	*N*	% missing	Range	Mean(SD)
BBTS Before Age 18	360	.28	12–36	17.84 (4.62)*
BBTS After Age 18	355	.53	12–31	16.37 (3.72)*
CAPE Positive subscale (symptoms)	362	.30	20–75	42.05(11.41)*
CAPE Positive subscale (distress)	362	0	20–70	37.89 (13.07)
DES-II	383	0	0–100	38.85(22.66)*
PAM-R Disorganised scale	364	0	0–3	1.47(.78)
PTI-ASS Unresolved scale	157	0	1–3.5	1.5 (.58)*

^*^Data positively skewed; interquartile range (for skewed data): BBTS before age 18 = 7; BBTS after age 18 = 4; CAPE: 15.7; DES-II: 36.43; PTI-ASS = .83

### Structural validity

Factor loadings of individual items are presented in Table 3, alongside Pollard et al.’s [38] EFA results. All items loaded onto the expected factors; all item loadings were > 0.4 [59]. The three-factor model based on Pollard et al.’s [38] research met the criteria for the CFI (0.955), TLI (0.950), and GFI (0.956). It was noted, however, that the three-factor model did not meet the RMSEA criteria (RMSEA = 0.107). Individual items were, therefore, re-evaluated according to the attachment literature, and modification indices, expected parameter change, and the standardised residual covariance matrix were examined for potential sources of misfit. Four items (9, 13, 15, and 22) were identified as potentially describing features of attachment which were not unique to the target attachment style and may be contributing to misspecification. As an experiment, these items were removed and CFA was conducted on the revised model—RMSEA was adequate (0.077), whilst the remaining three indices (CFI = 0.978; TLI = 0.975; GFI = 0.976) indicated a “good” model fit [58]. However, given that RMSEA can be influenced by sample size and all items loaded onto hypothesised factors above 0.4, all items were retained for subsequent analyses.

**Table 3:** PAM-R item factor loadings from the CFA and Pollard et al.’s [38] EFA

Factor and item	Factor loading (present study)	Factor loading (Pollard et al. [38])
Avoidant		
(1) I prefer not to let other people know my “true” thoughts and feelings	.724	.474
(2) I find it easy to depend on other people for support with problems or difficult situations (RS)	.723	.665
(6) I usually discuss my problems and concerns with other people (RS)	.703	.678
(11) I find it difficult to accept help from other people when I have problems or difficulties	.779	.500
(13) It helps to turn to other people when I’m stressed (RS)	.569	.627
(19) I try to cope with stressful situations on my own	.696	.536
Disorganised		
(2) I find close relationships overwhelming	.771	.739
(4) I feel frightened in close relationships	.834	.796
(8) I find people I am in close relationships with to be unpredictable in their actions and behaviours	.453	.439
(12) When I try to get close to someone sometimes I shut down and find it difficult to think or move	.789	.761
(15) Sometimes I am confused by my feelings towards others	.750	.553
(17) I want close relationships, but being close makes me feel frightened	.897	.626
(18) I often freeze when I try to get close to someone	.821	.627
(21) I want to be close to others but I often find myself pulling away when I am	.802	.578
(23) When I form close relationships I lose sense of who I am	.585	.487
Anxious		
(5) I tend to get upset, anxious, or angry if other people are not there when I need them	.575	.740
(7) I worry that key people in my life won’t be around in the future	.629	.519
(9) I ask other people to reassure me that they care about me	.433	.563
(10) If other people disapprove of something I do, I get very upset	.637	.580
(14) I worry that if other people get to know me better, they won’t like me	.925	.509
(16) I worry a lot about my relationships with other people	.796	.561
(20) I worry if I displease other people, they won’t want to know me anymore	.851	.764
(22) I worry about having to cope with problems and difficult situations on my own	.573	.632

*CFA* Confirmatory Factor Analysis, *EFA* Exploratory Factor Analysis, *PAM-R* Psychosis Attachment Measure-Revised, *RS* Reverse scored

### Reliability

#### Internal consistency

Cronbach’s alpha for the three subscales of the PAM-R was as follows: Avoidant = 0.804, Anxious = 0.845, and Disorganised = 0.887. Alphas if deleted statistics exceeded 0.770 suggesting that all individual items were relevant to their respective scales.

#### Test–retest reliability

Fifty-nine participants from this study provided responses to the PAM-R at Time 1 and Time 2. ICCs (absolute agreement, two-way mixed-effects model) with 95% Confidence Intervals: Avoidant = 0.863 (0.770–0.918), Anxious = 0.957 (0.927–0.974), and Disorganised = 0.910 (0.839–0.948).

#### Construct validity

Correlations and significance values are presented in Tables 4 and 5. The disorganised subscale had a large positive correlation with the fearful subscale of the RQ, and a moderate positive correlation with the unresolved scale of the PTI-ASS. Moderate positive correlations were also observed between the disorganised subscale and the frequency of positive symptoms, and the level of distress associated with these symptoms, as measured by the CAPE-42 Positive Symptoms subscale. Similarly, the PAM-R disorganised factor was moderately positively correlated with the DES-II, which captured dissociative experiences. Responses on the BBTS were grouped according to the level of betrayal (high, medium, and low) and whether the traumatic experience happened before or after 18 (Table 5). There was a small, but highly significant, positive correlation between the disorganised subscale and all BBTS groups except for the “low betrayal” group after aged 18. Traumatic experiences within each betrayal group had stronger associations with the disorganised subscale if they occurred before the age of 18. “High betrayal” items before aged 18 were most strongly associated with the disorganised subscale (*r* = 0.275)—the observed correlations were progressively weaker across the lower betrayal group. This pattern of correlations was also observed in the “after 18” groups. Compared to the other PAM-R subscales, the strongest correlations within each group were found for the disorganised scale.

**Table 4:** Correlations between the PAM-R disorganised factor and the CAPE-42, DES-II, PTI-ASS Unresolved scale, and the RQ Fearful factor

Measure	Correlation
CAPE positive symptoms (frequency)	.457**
CAPE positive symptoms (distress)	.386**
DES-II total	.430**
PTI-ASS unresolved	.388**
RQ fearful	.512**

Correlations are Spearman’s rank-order; ** *p* =  < .001 All correlations are derived from the CAPE: Community Assessment of Psychic Experiences; DES-II: Dissociative Experiences Scale; PTI-ASS: Psychological Treatment Inventory-Attachment Style Scale; RQ: Relationship Question

**Table 5:** Correlations between the PAM-R disorganised subscale and BBTS (grouped by age and level of betrayal)

	BBTS Scale and Grouping					
PAM-R subscale	HB Before 18	HB After 18	MB Before 18	MB After 18	LB Before 18	LB After 18
Disorganised	.275**	.180**	.213**	.147**	.167**	.086
Anxious	.180**	.164**	.170**	.078	.076	.025
Avoidant	.170**	.069	.167**	.036	.145**	.060

Correlations are Spearman’s rank-order; ** *p* =  < .001; HB = high betrayal; MB = medium betrayal; LB = low betrayal

## Discussion

The CFA provided evidence for a three-factor model of the PAM-R (hypothesis 1), which included avoidant, anxious, and disorganised factors. Replication of Pollard et al.’s suggested factor structure resulted in an adequate-good fit across three out of the four fit indices. Further refinement of this model was achieved through removal of four items, which led to a “good” fit according to the CFI, TLI, and GFI, and “adequate” fit according to the RMSEA. Hypotheses 2 and 3 were also met—internal consistency and test–retest reliability exceeded the critical cut-offs for all three subscales using all the items. Notably, the disorganised subscale demonstrated “excellent” test–retest reliability across the 2-week timescale. This was in line with Pollard et al.’s. [38] findings; in their study, ICCs exceeded 0.823 and Cronbach’s alphas were greater than 0.791 across for all scales.

The disorganised subscale correlated in the expected directions with other related measures (hypotheses 4 and 5). However, a moderate correlation was found between the disorganised factor and the unresolved subscale of the PTI-ASS, whilst a large positive relationship was observed with the fearful subscale of the RQ. These findings suggest that the PAM-R may be more closely related to the fearful attachment captured by the RQ compared to disorganised attachment captured by the PTI-ASS. Nonetheless, the disorganised scale was not perfectly correlated with the RQ, perhaps because the RQ describes disorganised/fearful attachment in a single paragraph based on two dimensions: dependence and avoidance of intimacy [52]. Although these are core features of the disorganised PAM-R scale, the PAM-R also includes items relating to confusion, disorientation, and freezing—experiences which are characteristic of this attachment style according to both the child and adult literature [33, 34]. Similarly, the moderate correlation with the PTI-ASS may be explained by the fact that the PTI-ASS was designed with romantic, intimate relationships in mind [39], and may be less accessible to the target population given that these individuals are at greater risk of being socially isolated [5]. In addition, PTI-ASS’ unresolved items predominantly target aggression and mistreatment within relationships, as opposed to the emotional experience of the individual, which is a central focus of the PAM-R.

A moderate, highly significant, association was observed between the Disorganised PAM-R scale and measures of dissociative experiences and positive psychotic symptoms. This supports previous studies which have reported associations between these constructs and the disorganised attachment style [35, 36]. A small, positive relationship was established between with disorganised factor and the measure of trauma (BBTS); the magnitude of this correlation fell somewhat below expectations and Pollard et al.’s [41] findings (*r* > 0.3 for both age groups). This may in part be explained by the fact that the data for the BBTS were positively skewed, indicating that most participants had experienced one or more traumatic events “once or twice”. As this study did not collect information about potential protective factors, such as close relationships, supportive communities, and positive coping strategies [60], it is possible that impact of infrequent traumatic events was offset by significant positive and/or reparative experiences. However, it is noted that more significant abuse histories (as denoted by the “high” and “medium” betrayal groups of the BBTS) were more strongly associated with the disorganised factor, compared to the anxious or avoidant subscales. This is in line with previous research (e.g., [37]), which indicates that disorganised attachment is associated with higher proportions of interpersonal trauma.

### Strengths and limitations

The COSMIN criteria for good measurement properties [42] informed the development of study hypotheses and the statistical methods employed. As such, the results were evaluated against globally recognised standards that indicate whether a measure is “good enough” to be used in research or clinical settings. With respects to the sample, men and women were both well represented, as were varied self-reported psychiatric diagnoses. Despite the research team being based in the UK, almost 60% of participants were not British, which highlights the potential for the PAM-R to be used beyond its country of origin. It was noted that over 53% of the sample identified as LGBTQ +. Arguably people may have felt more comfortable reporting their LGBTQ + status online compared to in face-to-face research, but the proportion of our sample within this group far exceeds general population estimates in the US and UK, which are less than 10% [61, 62]. As the LGBTQ + community is both underserved within research, and reportedly at heightened risk of developing mental health issues [63], there is value in the relative over-representation of this group in the present sample. However, the authors recognise that there may be some developmental and interpersonal experiences which are relatively unique to and/or ubiquitous within the LGBTQ + community which may impact upon attachment behaviour [64], thus limiting the generalisability of this study. It is possible that advert for our study was distributed disproportionately to groups representing people from LGBTQ + communities, but to the best of our knowledge, our recruitment strategy was not different from other online studies conducted by members of our research group.

A further limitation of this study was that the majority of the sample (> 83%) identified as white. Although this was broadly in line with data from the UK and US censuses [65, 66], the absolute number of participants from other ethnic groups, particularly the black community, was very low. As several studies have indicated that the weighted prevalence of psychosis is much higher amongst the black population (e.g., [67, 68]) compared to white communities in the UK, it would be important for this limitation to be addressed in future research to ensure that the PAM-R is accessible and appropriate for a range of ethnic groups. This study also relied on self-reported experiences of psychosis and approximately 7% of the sample did not report a diagnosis of psychiatric diagnosis. Whilst a substantial majority (> 65%) described previous inpatient experience and clinically significant levels of dissociation, the risk that these findings may not generalise to a clinical setting, where the original PAM was tested, is noted. In addition, the online methodology and merged data set created a risk of participants completing the questionnaires more than once. However, as the projects were advertised via independent social media accounts, led by different researchers, and had recruitment windows that were approximately 2 years apart, it is argued that their respective reach and networks would have likely been sufficiently different, so that risk of repeat responders was not significant. Finally, in terms of the psychometric properties assessed within this study, discriminant validity was one key area which was not explored.

### Implications and future research

This study provided further validation of the PAM-R and evidence of its psychometric properties. The PAM-R demonstrated a theoretically relevant factor structure, and all subscales were found to internally consistent and stable over a two-week period. The new disorganised scale correlated in expected directions with other conceptually relevant measures, providing evidence for its concurrent and convergent validity. Together, these findings suggest that the PAM-R has good psychometric properties, giving researchers and clinicians alike access to a time- and cost-effective measure of attachment for individuals with experiences of psychosis. They also further contribute to the research base which indicates a relationship between disorganised attachment and positive psychotic symptoms, dissociation, and trauma. The authors tentatively suggest that the PAM-R may be further improved through the removal or adaptation of four items; however, this assertion would need to be confirmed within a future representative sample. Future research may usefully focus on validating the PAM-R in clinical settings. Kvrgic and colleagues [69] conducted a similar study with the German version of the original PAM; their findings could be extended through the inclusion of the disorganised subscale in an English-speaking population. The inclusion of a non-clinical population for comparison would also allow for the discriminant validity of the PAM-R to be examined. Separately, one of the key motivations for developing the PAM was the lack of valid attachment measures which did not focus on intimate or romantic relationships, as these were argued to be less accessible and less relevant for individuals with psychosis who are more at risk of experiencing interpersonal difficulties [5]. It is noted that individuals who are neurodivergent or experience other significant mental health issues (e.g., people diagnosed with personality or bipolar disorders) can have similar difficulties in in maintaining social connections, and thus, there is scope for future studies to investigate the application of the PAM-R in other potentially socially isolated groups.

## Conclusions

This study suggests that the PAM-R is a reliable and valid measure of attachment in psychosis. The findings provide further evidence of its structural dimensionality, internal consistency, test–retest reliability, and convergent validity. A three-factor solution was demonstrated by CFA; however, the authors propose that four of the items which may have contributed to the poor RMSEA statistic require further investigation. Future research is needed to replicate these findings within a representative clinical population and to establish discriminant validity.

## Supplementary Information

Below is the link to the electronic supplementary material.

Supplementary file1 (DOCX 17 KB)

## Data Availability

The data that support the findings of this study are available from the corresponding author upon reasonable request.
