# Supplementary material for: The revised Psychosis Attachment Measure: further psychometric evidence
Source: Soc Psychiatry Psychiatr Epidemiol. 2024 Mar 19;59(10):1803–13. doi: 10.1007/s00127-024-02624-2 (PMC11464540; doi:10.1007/s00127-024-02624-2)
Supplement: Supplementary file 1 — Supplementary file1 (DOCX 17 KB) [file 127_2024_2624_MOESM1_ESM.docx]

**Revised – Psychosis Attachment Measure (R-PAM)**

We all differ in how we relate to other people. This questionnaire lists different thoughts, feelings and ways of behaving in relationships with others.

Thinking generally about how you relate to other key people in your life, **please use a tick to show how much each statement is like you**. Key people could include family members, friends, partner or mental health workers.

There are no right or wrong answers

| Item | **Not at all** | **A little** | **Quite a bit** | **Very much** |
| --- | --- | --- | --- | --- |
| 1. I prefer not to let other people know my ‘true’ thoughts and feelings |  |  |  |  |
| 2. I find close relationships overwhelming |  |  |  |  |
| 3. I find it easy to depend on other people for support with problems or difficult situations |  |  |  |  |
| 4. I feel frightened in close relationships |  |  |  |  |
| 5. I tend to get upset, anxious or angry if other people are not there when I need them |  |  |  |  |
| 6. I usually discuss my problems and concerns with other people |  |  |  |  |
| 7. I worry that key people in my life won’t be around in the future |  |  |  |  |
| 8. I find people I am in close relationships with to be unpredictable in their actions and behaviours |  |  |  |  |
| 9. I ask other people to reassure me that they care about me |  |  |  |  |
| 10. If other people disapprove of something I do, I get very upset |  |  |  |  |
| 11. I find it difficult to accept help from other people when I have problems or difficulties |  |  |  |  |
| 12. When I try to get close to someone, sometimes I shut down and find it difficult to think or move |  |  |  |  |
| 13. It helps to turn to other people when I’m stressed |  |  |  |  |
| 14. I worry that if other people get to know me better, they won’t like me |  |  |  |  |
| 15. Sometimes I am confused by my feelings towards others |  |  |  |  |
| 16. I worry a lot about my relationships with other people |  |  |  |  |
| 17. I want close relationships, but being close makes me feel frightened |  |  |  |  |
| 18. I often freeze when I try to get close to someone |  |  |  |  |
| 19. I try to cope with stressful situations on my own |  |  |  |  |
| 20. I worry that if I displease other people, they won’t want to know me anymore |  |  |  |  |
| 21. I want to be close to others but I often find myself pulling away when I am |  |  |  |  |
| 22. I worry about having to cope with problems and difficult situations on my own |  |  |  |  |
| 23. When I form close relationships, I lose sense of who I am |  |  |  |  |
